# A Screening Tool for the Direct Analysis of Marine and Freshwater Phycotoxins in Organic SPATT Extracts from the Chesapeake Bay

**DOI:** 10.3390/toxins12050322

**Published:** 2020-05-13

**Authors:** Michelle D. Onofrio, Claude R. Mallet, Allen R. Place, Juliette L. Smith

**Affiliations:** 1 Virginia Institute of Marine Science, College of William & Mary, Gloucester Point, VA 23062, USA; mdonofrio@email.wm.edu; 2 Separation Technologies, Waters Corporation, Milford, MA 01757, USA; claude_mallet@waters.com; 3 Institute of Marine and Environmental Technology, University of Maryland Center for Marine and Environmental Sciences, Baltimore, MD 21202, USA; place@umces.edu

**Keywords:** phycotoxins, mass spectrometry, UPLC-MS/MS, at-column dilution, SPATT, microcystins, okadaic acid, azaspiracids, pectenotoxins, brevetoxins

## Abstract

Many detection methods for phycotoxins, bioactive compounds produced by harmful algae, focus on one compound or a class of related compounds. Multiple harmful algal species often co-occur in the environment, however, emphasizing the need to analyze for the presence of multiple groups of marine and freshwater phycotoxins in environmental samples, e.g., extracts from solid phase adsorption toxin tracking (SPATT). Two methods were developed to screen for 13 phycotoxins (microcystin-RR, -LR, -YR, azaspiracid-1, -2, karlotoxin 3, goniodomin A, brevetoxin-2, yessotoxin, pectenotoxin-2, dinophysistoxin-1, -2, and okadaic acid) in organic SPATT extracts using ultra-performance liquid chromatography–tandem mass spectrometry (UPLC-MS/MS) equipped with a trapping dimension (trap) and at-column dilution (ACD). The performance of each compound under 36 combinations of chromatographic conditions was characterized, and two final methods, acidic and basic, were selected based on peak shapes, signal intensities, resolution, and the separation in time of positive and negative MS ionization modes. Injection volumes of up to 1 mL were possible through trap/ACD technology, resulting in limits of detection between 0.001 and 0.05 µg/L across the analytes. Benefits highlighted in this study, beyond the improved detection limits and co-detection of multiple toxin groups, include the ability to inject samples of 100% organic solvent, ensuring analyte stability and streamlining workflow through the elimination of laborious sample preparation steps.

## 1. Introduction

Bioactive compounds produced by various species of harmful algae, or phycotoxins, have the potential to cause both mass mortalities of wildlife [1,2] and severe illnesses in humans [2,3]. Animals that ingest algae associated with phycotoxins may experience direct health effects and/or mortality, or they may serve as vectors of exposure to other organisms when consumed (Table 1) [3,4]. Human exposure can occur through the consumption of contaminated seafood [3] or drinking water [5], or through respiration of aerosolized compounds [6]. Due to their hazardous effects, phycotoxins are monitored in edible tissue, drinking water, and the environment.

While robust and sensitive mass spectrometry methods have been incorporated into some monitoring programs, these methods are often developed for a single group of compounds, especially in regions where annual blooms of the same species occur. The global expansion in range and frequency of harmful algal blooms [7,8,9], however, emphasizes the need for multi-toxin detection methods for co-occurring harmful algae. Research regarding the presence of co-occurring harmful algal species and/or their associated phycotoxins has recently expanded, and such co-occurrences have now been documented globally [10,11,12,13,14,15,16]. Some algal species can co-produce multiple phycotoxins (Table 1), further adding to the suite of compounds that may be present in a given environmental sample. Additionally, the salinity gradient in estuaries and along coastal zones, such as Chesapeake Bay and its Eastern Shore, presents an increased probability for the co-occurrence of phycotoxins associated with marine and freshwater species [4,16,17].

A limited number of LC-MS methods for the co-detection and quantification of marine and freshwater phycotoxins have been published [18,19,20,21]. We sought, however, to develop a multi-toxin screening method for Chesapeake Bay’s unique suite of toxins observed across the salinity gradient (Table 1), streamline workflow, and improve method performance required for their trace analysis.

**Figure 1:**
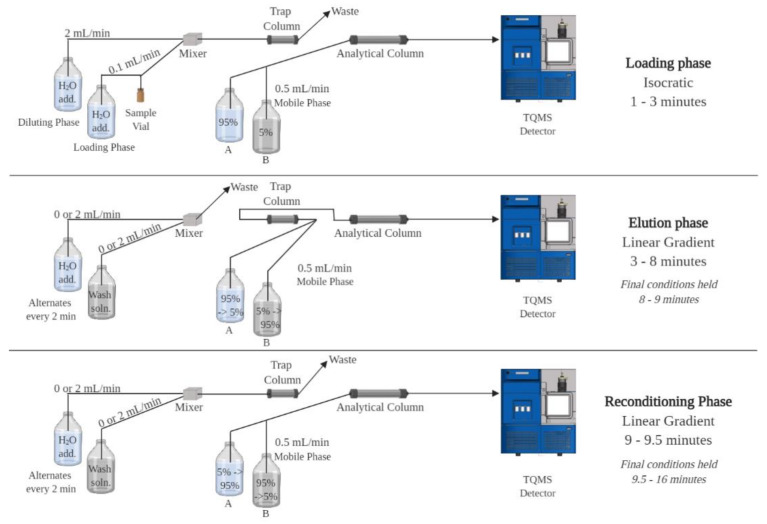
Schematic of a UPLC-MS/MS system equipped with a trapping dimension (trap) and at-column dilution (ACD).

Ultra-performance liquid chromatography with tandem mass spectrometry (UPLC-MS/MS) equipped with a trapping dimension (trap) and at-column dilution (ACD) (Figure 1) was investigated. Applying trap/ACD to UPLC-MS/MS allows for large volume injections (50–1000 µL) of extracts in 100% organic solvent [22]. This technology, therefore, has the potential to facilitate trace level analysis by improving detection limits, removes the need for dry-down and concentration steps, streamlining sample preparation [22], and minimizes lipophilic phycotoxin degradation or sorption loss that would otherwise occur in more typical aqueous extracts. As the intention is to later pair this novel screening method with SPATT, a common passive sampling tool that accumulates phycotoxins over deployment [11,23,24,25,26,27], a developed method also requires evaluation using SPATT samples. By applying UPLC-MS/MS with trap/ACD to SPATT organic extracts we expect to combine the benefits of low detection limits with cumulative sorption in the environment, respectively.

The overarching goal of this study, therefore, was to develop a screening tool for the detection of multiple marine and freshwater phycotoxins in SPATT samples collected from Chesapeake Bay. This study also aimed to conduct a comprehensive investigation of phycotoxin behavior under a variety of column and mobile phase chemistries. Phycotoxins included in this study (Table 1) were chosen to represent toxins (1) spanning a range of hydrophobicities from highly lipophilic (PbTx-2) to more polar (KmTx 3, microcystins), (2) federally regulated in marine seafood products or freshwater systems (i.e., OA, DTXs, microcystins), (3) of emerging national interest (AZAs), and/or (4) associated with animal illness within the local Chesapeake Bay (i.e., karlotoxins, goniodomin A). All variants within each phycotoxin group were not included in this study; instead, selection amongst congeners focused on parent structures or congeners most commonly detected in U.S. waters. For field-sample analysis, SPATTs deployed in the Chesapeake Bay region were extracted and analyzed for endogenous toxins and matrix effects using the finalized methods.

## 2. Results and Discussion

The selected marine and freshwater phycotoxins, ranging in hydrophobicity, mode of toxicity, and molecular weight (Table 1), were assessed for their performance under multiple combinations of chromatographic conditions in the development of a screening tool for Chesapeake Bay. The 13 diverse compounds were successfully co-detected in the same organic extract using two optimized final methods, one using acidic loading, diluting, and mobile phase solutions, and another using basic loading, diluting, and mobile phase solutions, with a C18 trap and analytical column. Method development was conducted using 50 µL injections of mixed toxins in 100% organic solvent to allow for the co-detection of trace compounds, improve analyte stability, and streamline workflow. The optimized method was then applied to 1000 µL injections as a proof-of-concept, demonstrating that the method could perform under circumstances where further-improved detection was required. The injection of elevated volumes, 50–1000 µL, of organic solution directly onto the UPLC-MS/MS system was possible via the trap/ACD technology. These final methods may be further adapted to include additional compounds of interest and other types of environmental samples beyond SPATTs.

### 2.1. Chromatographic Method Development

**Figure 2:**
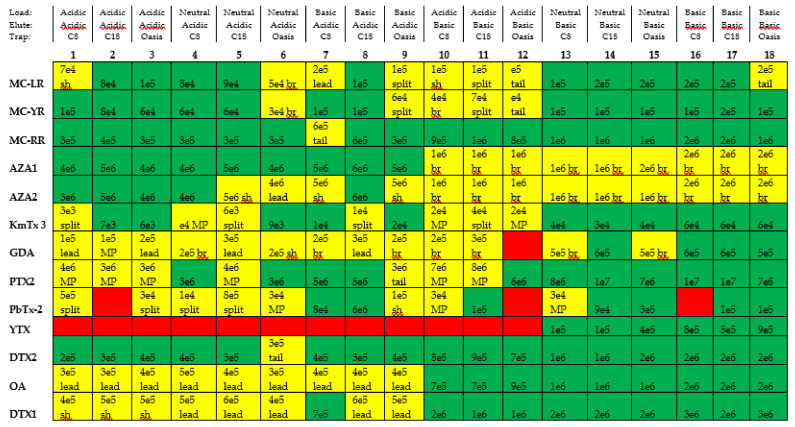
Method development results showing the performance of 13 phycotoxins in 18 of the 36 trialed combinations of chromatographic conditions using a C18 analytical column. Loading phases were varied between acidic (formic acid), neutral (no additive), and basic (ammonium hydroxide); elution mobile phases were varied between acidic (formic acid) and basic (ammonium hydroxide), and C8, C18, and Oasis HLB Direct Connect HP trap columns were tested. Loading phases contained ultrapure water with 5% additive, while elution mobile phases contained (A) ultrapure water and (B) acetonitrile with 0.5% additive. Green boxes represent uniform, gaussian peak shape. Yellow boxes represent poor peak shape (sh: shoulder, br: broad, lead: leading, tail: tailing, MP: multiple peaks, split: split peak). Red boxes represent no detection. Numbers represent peak height. These results were used to devise the final methods listed in Table 2, which include modifications to methods 2 and 17.

The performance of the 13 selected compounds in 36 combinations of trapping columns, analytical columns, and mobile phases was evaluated and characterized during method development (Figure 2). Overall, the C18 analytical column produced higher signal intensities than the C8 analytical column under acidic conditions, and signal intensities were comparable under basic conditions (data not shown). The C18 analytical column was, therefore, chosen for both methods with the added benefit that no changing of columns was needed between subsequent runs. The performance of C18 and Oasis Direct Connect trapping columns were superior to C8 in most cases (Figure 2). Coupling low loading pH with high eluting pH, or vice versa, produced poor peak shapes and relatively low signal intensities for many compounds evaluated. MC-RR, MC-LR, and MC-YR performed well under both basic and acidic conditions, but exhibited the highest sensitivity using basic conditions. AZA1 and AZA2 were best detected under acidic conditions and produced broad peaks under basic conditions. Under acidic conditions, a small peak with the same confirmatory MRM transition as PTX2 was detected at a retention time of 7.17 min, shortly after the peak for PTX2 at 6.76 min, while only one peak at 6.76 min was present for PTX2 under basic conditions. This agrees with published results about the epimerization of PTX2 under acidic conditions [30]. YTX was undetectable under acidic conditions but performed well under basic conditions. PbTx-2 and GDA produced better peak shapes and signal intensities under basic conditions than acidic conditions. DTX2 was detectable and exhibited good peak shape under almost all tested conditions, but had the greatest sensitivity using basic conditions. OA and DTX1 behaved similarly, with best peak shape and sensitivity observed under basic conditions. KmTx 3 exhibited the best sensitivity under basic conditions, but produced acceptable peak shape and sensitivity under acidic conditions with the C18 and Oasis trapping columns.

Two methods, an acidic and a basic, were necessary for the detection of all 13 compounds evaluated. Although some compounds performed well in multiple combinations of chromatographic conditions, Method 2 (acidic conditions) and Method 17 (basic conditions) were chosen as the best methods to optimize based on each compound’s peak shape and signal intensity, resolution between adjacent peaks, and the separation in time of positive and negative ionization modes (Figure 2). Retention time windows were used to eliminate loss in sensitivity from rapid switching between ESI+ and ESI− [31]. The range of retention times for compounds evaluated in ESI− (OA, DTX1, DTX2, YTX), therefore, had to be separated from the rest of the compounds. To satisfy this condition, MC-RR, -YR, and -LR were analyzed under acidic conditions even though they exhibited higher sensitivity when using basic conditions (Figure 2). This compromise ensured a retention time window for OA, DTX1, DTX2, and YTX that did not overlap with any positively ionizing compounds. When using the basic method, the mass spectrometer operates in ESI− from 3–6.20 min, and in ESI+ from 6.20–8 min. All compounds included in the acidic method were detected using ESI+.

### 2.2. Final Chromatography Methods

Once the compounds were designated to their acidic or basic method during development (Figure 2), steps were taken to further optimize the chromatographic conditions to achieve two final methods (Table 2). The compositions of the loading and mobile phases were refined from developmental methods by varying the amount (strength) of additive and by adding ammonium formate as a buffer to the acidic loading, diluting, and mobile phases. Overall, higher-strength additive loading (5%) and mobile (0.5%) phases (Figure 2) were adequate for the detection of all compounds tested. Lower-strength additive solutions, however, were selected for method optimization as they produced the highest sensitivity and eliminated shifts in retention time between injections. Final acidic loading and mobile phase solutions contained 50 mM (0.2%) FA + 2 mM ammonium formate, and final basic loading and mobile phases solutions contained 6.7 mM (0.1%) ammonium hydroxide (Table 2).

**Figure 3:**
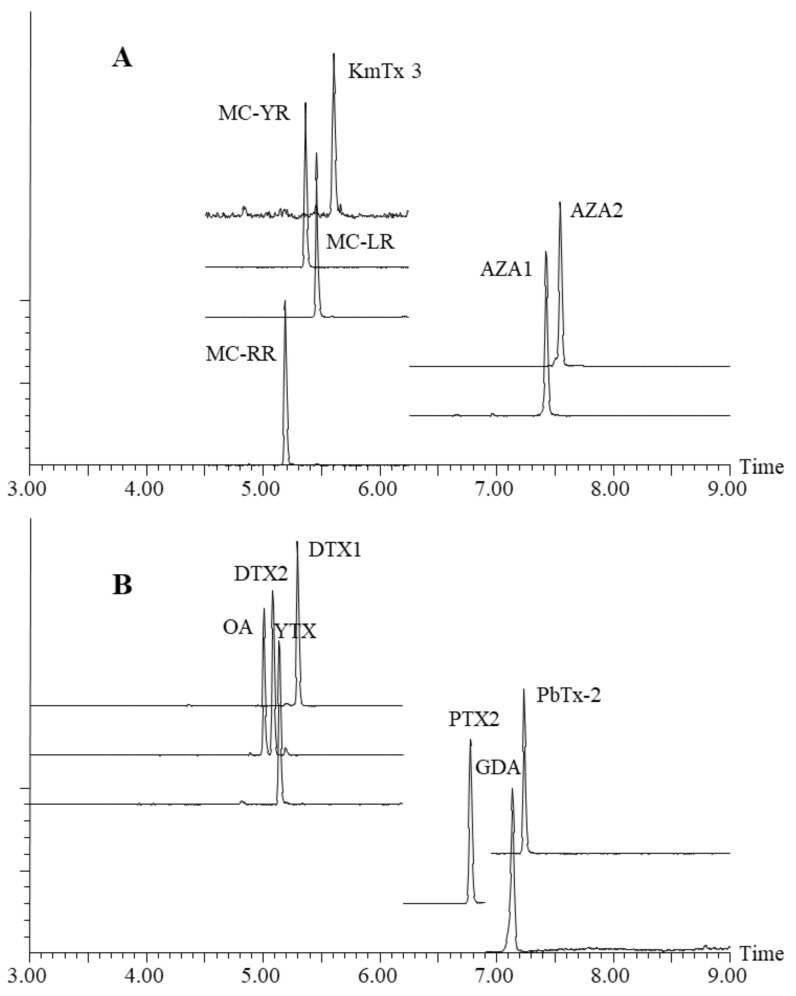
Chromatograms obtained from a 50 µL mixed-toxin standard run of the final, optimized methods under (**A**) acidic conditions, and (**B**) basic conditions.

Using the two final methods, carryover and other performance metrics were evaluated, and field-sample analysis was conducted (see Section 2.4 and Section 2.5); ultimately all compounds were well resolved and exhibited acceptable peak shape and signal intensity (Figure 3).

### 2.3. Reduction of Carryover

Upon the first attempt at evaluating the performance of the final methods (Table 2), carryover between samples resulted in high %RSD values (up to 26%) as peak areas for each compound increased during triplicate 50 µL injections from the same vial. To reduce carryover, however, a wash step was introduced into the method, and was applied to all instrumental components up to, but not including, the analytical column: i.e., the injection and trapping circuits are washed with the solution. A wash solution containing acetonitrile, methanol, isopropyl alcohol, and acetone (1:1:1:1) + 5% formic acid was applied through the chromatographic inlet method at a flow rate of 2 mL/min in 2 min intervals, alternating with the aqueous loading phase. While the mobile phase gradient ran through the trapping and analytical columns between 3 and 8 min, the wash intervals ran through the flow-through needle injection loop and associated circuit, being sent to waste before reaching the trapping column. At 9 min, the wash intervals extended into the trapping column and were sent to waste without reaching the analytical column. The addition of these wash steps reduced carryover from 18.6% to <4%, and extended the run time from 10 to 16 min, producing a suitable wash step to move forward as part of the final two methods.

### 2.4. Method Performance Characteristics

To evaluate method performance characteristics, acidic and basic methods were performed sequentially. All acidic injections were run first, followed by all basic injections. Three blank injections using the basic method were included after the last acidic injection and before the first basic injection to equilibrate the instrument to basic conditions.

Once carryover was addressed, linearity and percent relative standard deviation (%RSD) were evaluated and determined acceptable for the final two methods (Table 3). More specifically, two 10-point standard curves, across multiple orders of magnitude, were produced in triplicate for each compound to assess linearity across multiple orders of magnitude. One standard curve ranged from the closest concentration to each LOD (Table 3) through 5 µg/L, and the other from 5 µg/L through 50 µg/L. Linear regressions were performed on each curve, and all curves were linear within the tested range with squared correlation coefficients (R^2^) values > 0.99. Similarly, to determine repeatability, percent relative standard deviation (%RSD) was used to compare peak areas obtained from replicate injections of the same mixed-standard solution. All %RSD values were below 5%, with the exception of PbTx-2 at 7.18% (Table 3). These results indicate that the final methods are reproducible for all 13 phycotoxins.

Limits of detection (LOD) and quantitation (LOQ) were determined for high-volume injections: 50 and 1000 µL (Table 3). Using standard solutions, LODs generated with 50-µL injections were, in general, on par with previously-reported LODs using traditional UPLC-MS/MS methods, with the exception of microcystins for which improved LODs have been reported (Table 3). By further increasing the injection volume to 1000 µL, lower limits of detection (LOD) were achieved for all 13 phycotoxins, surpassing published LODs for traditional UPLC-MS/MS analysis (Table 3). All experimentally-derived LODs (S/N ≥ 3) were confirmed using triplicate injections of a mixed-standard solution at the LOD concentration, and/or through their comparison to mathematically-derived LODs (Table 3). In contrast to some literature methods, the methods reported herein did not require the addition of a concentration step, e.g., solid-phase extraction (SPE), evaporation to dryness, or the use of a high-resolution mass spectrometer to achieve improved detection limits (Table 3). 

Together these results suggest that high-volume injections, up to 1000 µL, may be used to improve detection of analytes in field samples. Higher injection volumes are only recommended when trace analysis is needed, however, as instrument fouling can occur more rapidly when injecting these high volumes. Blank injections included after 1000 µL injections confirmed the lack of additional carryover due to the increased injection volume. We also note that repeatability was not sacrificed with high injection volumes, as %RSD values for both the 50 µL and 1 mL injections were within acceptable limits (Table 3).

This application highlights the benefits of UPLC-MS/MS with trap/ACD technology. Sample preparation time and labor were reduced, and evaporative loss was eliminated as 100% organic extracts were analyzed directly without additional clean-up or concentration and reconstitution steps. The reported low detection limits will be useful in exploring the prevalence of trace phycotoxin concentrations present in the Bay. This technology is not limited to phycotoxins, and application for other groups of compounds typically analyzed by LC-MS is possible.

### 2.5. SPATT Analysis

**Figure 4:**
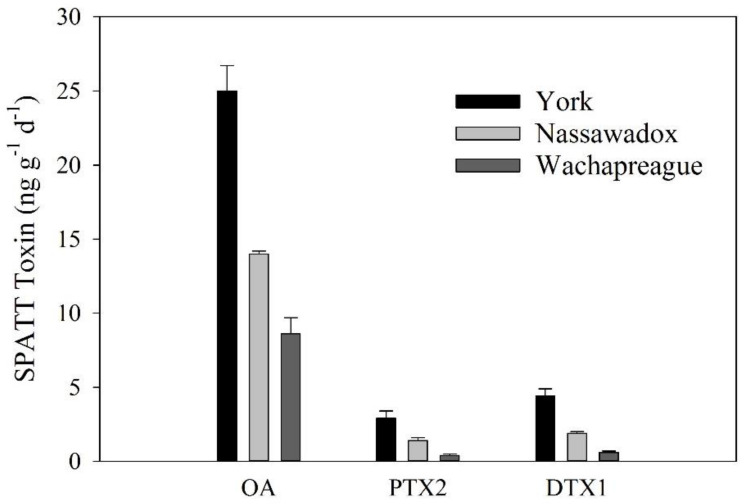
Endogenous OA, PTX2, and DTX1 in SPATT extracts from three sites in the lower Chesapeake Bay region using 50-µL injections under the final two methods: acidic and basic. Data were generated using the parent > parent transitions and are represented as ng toxin per gram SPATT resin per day, and error bars represent standard deviation from triplicate field samples.

Triplicate SPATTs deployed at three sites in the Chesapeake Bay region were extracted using traditional methods [23], and 50 µL of organic extract was analyzed by the two final, optimized analytical methods (Table 2). In every SPATT extract that was analyzed, endogenous OA, DTX1, and PTX2 were detected (Figure 4). The amount of endogenous toxins in SPATT extracts were similar whether calculated using parent > parent or parent > daughter transitions (Table 4). SPATT extracts from the York River had the highest levels of each of these phycotoxins, while SPATT extracts from Wachapreague had the lowest. Across sites, OA (8.6–25 ng OA/g resin/day) was highest relative to DTX1 (0.06–4.4 ng DTX1/g resin/day) and PTX2 (0.4–2.9 ng PTX2/g resin/day). The positive detection of these phycotoxins confirms that this method is suitable for use as a screening tool for SPATT extracts.

Furthermore, when these SPATT extracts were spiked with a multi-toxin solution, all 13 toxins were detectable with S/N > 3 for the parent > parent transitions, showing that the final methods are suitable as a screening tool across the diverse suite of marine and freshwater toxins in Chesapeake Bay and the complex matrix associated with SPATTs. Varying levels of signal enhancement and signal suppression, however, were observed for each compound (Table 5), suggesting that targeted cleanup, matrix-matched standard curves, or the standard addition method should be considered in studies with more quantitative objectives. Signal suppression or enhancement was calculated by comparing spiked samples to a standard solution using the equation *Suppression or Enhancement* (%) *= 100 × spiked extract peak area/spiked standard peak area.* Signal suppression was observed for MC-RR, -LR, and -YR, AZA1, and AZA2. YTX showed signal enhancement in Nassawadox and Wachapreague extracts. Because OA, DTX1, and PTX2 were already present in the SPATT extracts from all three sites, matrix effects for these three compounds were evaluated by comparing the measured amount of endogenous toxins in non-spiked injections to the measured amount of toxins in the spiked samples. Percent suppression or enhancement was calculated as *µg measured in spiked sample/(µg endogenous toxin + µg toxin added)×100*. 

Instrumental LODs presented herein (Table 3) were determined using calibration standards, and as such, toxin-specific matrix effects in SPATT extracts may result in modified limits of detection. More specifically, phycotoxins that show suppression could be expected to have higher LODs in matrix, while phycotoxins that show enhancement could result in lower LODs in matrix (Table 5). As presented here, these methods serve as a screening tool for multiple phycotoxins present in SPATT extracts, part of an explorative study to determine which phycotoxins are present in Chesapeake Bay. For strictly quantitative analyses of trace-level concentrations, however, the impact of matrix effects on limits of detection can be further explored, but matrix effects will likely vary based on season, system, salinity, and depth of sampling, as typical with estuarine environmental samples, and may also vary based on injection volume, e.g., 50 µL versus 1000 µL.

## 3. Conclusions

Two UPLC-MS/MS methods with trap/ACD were developed and optimized to encompass 13 phycotoxins of interest. The use of C18 trapping and analytical columns for both developed methods allows them to be run sequentially, without an exchange of hardware, resulting in the automated analysis of a single sample for all 13 phycotoxins. This technology also allows samples to be injected from solutions in 100% organic solvents, thereby increasing analyte stability and solubility. In practice, these benefits allow for organic extracts from SPATTs, or other environmental samples, to be injected directly without the need for dry-down and reconstitution steps, thus streamlining processing by eliminating hours, or even days, of sample preparation time and avoiding potential evaporative loss. Additionally, this work demonstrates proof-of-concept for the use of this instrumentation with high-volume injections. In practice, if results from a 50-µL injection show analyte(s) at or near the limit of detection/quantification, the sample can easily be rerun using a higher injection volume, up to 1 mL, to increase sensitivity instead of needing to concentrate the analyte(s) through further sample preparation. These methods were developed for use as a screening tool for the analysis of multiple marine and freshwater phycotoxins present in a single sample. For a strictly quantitative application of the developed methods, however, Figure 2 may be used as a reference to choose chromatographic conditions best suited for the specific suite of target compounds, further reduction of the matrix using additional sample clean-up steps could also be explored, and additional parent to daughter transitions could be included for quantification or further confirmation.

The selected compounds spanned marine and freshwater phycotoxins and a range of hydrophobicities relevant to Chesapeake Bay and other regions. These methods may be adapted to include congeners, other phycotoxins with similar polarities, or other classes of bioactive compounds traditionally analyzed by LC-MS. Figure 2 provides information about peak shape and relative signal intensity for the tested sets of chromatographic conditions, and can be used as a resource to select methods best suited for a particular group of phycotoxins. The ability to inject large volumes and screen for low concentrations of multiple marine and freshwater phycotoxins present in the environment is beneficial for both research and monitoring purposes. Detecting low concentrations may improve baseline data, allows for further research into the effects of low-level, chronic exposure, and provides the potential for early warning when phycotoxin concentrations begin to rise in samples collected for monitoring purposes. SPATT-based kinetic studies have not yet been completed for all phycotoxins included in our study. As such, we do not recommend that SPATTs replace more-traditional sampling methods in support of seafood or drinking water safety regulation, but rather serve as complementary sampling for monitoring purposes or research. Additionally, the intended use of these analytical methods is for research purposes; these methods are not recommended for use in regulatory settings without proper validation and certification.

## 4. Materials and Methods 

### 4.1. Reagents and Analytical Toxin Standards

All acetonitrile (ACN), methanol (MeOH), isopropyl alcohol (IPA), and acetone (ACT) used were LC-MS grade (Honeywell Burdick & Jackson, MI, USA). Ultrapure water was prepared using a Milli-Q system (Millipore, Billerica, MA, USA). Formic Acid Optima LC/MS (Thermo Fisher Scientific, Waltham, MA, USA), Ammonium Formate Optima LC/MS (Thermo Fisher Scientific, Waltham, MA, USA), and ammonium hydroxide eluent additive for LC-MS (≥25%, Honeywell Fluka, Charlotte, NC, USA) were used for mobile phases. Formic Acid 98% (EMD Millipore, Merck, Germany) was used for the carryover wash solution.

The following biotoxin certified reference materials were purchased from the National Research Council Canada: azaspiracid-1 (CRM-AZA1-b), azaspiracid-2 (CRM-AZA2-b), dinophysistoxin-1 (CRM-DTX1-b), dinophysistoxin-2 (CRM-DTX2-b), okadaic acid (CRM-OA-d), pectenotoxin-2, (CRM-PTX2-b), and yessotoxin (CRM-YTX-c). Brevetoxin-2 was purchased from Abcam (ab143469). A microcystin-RR, -YR, -LR mixed solution was purchased from Sigma Aldrich (33578-1ML). Karlotoxin 3 (KmTx 3) was purified from *Karlodinium veneficum* by Allen Place (UMCES, Maryland). Goniodomin A was purified from *Alexandrium monilatum* by Drs. Constance and Tom Harris [37] and provided by Dr. Kimberly Reece (VIMS).

### 4.2. Instrumentation

#### 4.2.1. Mass Spectrometry Conditions

A tandem quadrupole Xevo TQ MS (Waters, Milford, MA, USA) equipped with electrospray ionization (ESI) was used with multiple reaction monitoring (MRM) for detection. Capillary voltage was 3.00 kV, desolvation temperature was 450 °C, desolvation gas flow was 1100 L/hr, collision gas flow was 0.15 mL/min, and source temperature was 150 °C. The mass spectrometer was operated in both ESI+ and ESI− modes.

Before chromatography was evaluated, direct infusion experiments were performed on all 13 phycotoxins to optimize cone voltage values for each compound, and collision energy values were tested by evaluating the production of fragment (daughter) ions (Table 6). A concentrated solution (50–100 µg/L) of each compound was prepared in methanol. Each solution was individually introduced into the mass spectrometer through direct infusion. First, composite spectra across 30 MS scans were assessed for various cone voltage values, starting at 20V and increasing in 10V increments. The optimum cone voltage was determined as the cone voltage value that produced the highest molecular ion signal. Using the optimized cone voltage, composite spectra across 30 MS/MS scans were then assessed. Starting at 5 eV, collision energy was increased by 5 eV for each consecutive spectrum. From these spectra, daughter ions, and associated collision energies were evaluated. The collision energy values that resulted in the highest relative signal for the daughter mass compared to the parent mass were selected. Once these values were chosen, the exact mass for the daughter ion was chosen by observing the *m*/*z* value corresponding with the apex of the daughter ion peak. Parent > daughter transitions observed in this study resulted in a small loss of sensitivity due to poor fragmentation. To compensate for this loss in sensitivity, the parent > parent transition for each compound was used during method performance characterization, while the dominant parent > daughter transition was chosen for confirmation (Table 6). All parent > parent transitions were evaluated using a collision energy of 2 eV to minimize fragmentation.

All compounds were assessed in both positive and negative ionization modes, and the mode resulting in the highest signal was chosen (Table 6) for the subsequent chromatography experiments and evaluation of the final methods. Of the 13 compounds, most were best detected in ESI+ mode, however, four analytes (OA, DTX1, DTX2, YTX) were best detected by mass spectrometry using ESI−. Due to the limited availability of purified material, KmTx 3 did not undergo direct infusion, and literature values were instead used for the purpose of method development [36].

#### 4.2.2. Chromatographic Conditions

For all method development and method evaluation, the UPLC with trap/ACD system (Figure 1) comprised three binary pumps, one autosampler equipped with a 250 μL loop, and one column manager. The fluidics were set in a 2-1 trap/elute configuration. Acquity I class binary solvent managers (Waters, Milford, MA, USA) were used within the UPLC with trap/ACD system: for the loading flow, ACD, and elution. At-column dilution was applied at a 20:1 ratio of ACD flow rate with the dilutor stream set to 2 mL/min, and the loading flow rate set to 0.1 mL/min. The elution mobile phase flow was set to 0.5 mL/min. Loading and diluting phases were prepared with ultrapure water. Acidic loading and diluting phases were prepared with formic acid, basic with ammonium hydroxide, and neutral with no additives. Acidic and basic mobile phases were prepared with ACN for the organic (B), and ultrapure water for the aqueous (A), using the same additives as above. Injection volumes were 50 µL unless otherwise stated. The sample injection volume was carried by the loading flow, mixed with ACD flow, and sent to the trapping column. During the first 3 minutes of the 16-min chromatographic run, these loading conditions were applied while mobile phase flow was sent through the analytical column and to the mass spectrometer. This three-minute loading period is necessary for the loading circuit to empty the 250 µL loop, working in tandem with the diluting circuit to bring the full sample volume to the trapping column. At 3 min, valve positions were switched so that the mobile phase flowed through the trapping column before reaching the analytical column. This flow brings analytes to the analytical column, elutes them off the analytical column, and brings them to the mass spectrometer for detection. A linear gradient was applied to the mobile phase from 3–8 min, transitioning from 5% B to 95% B. From 8–9 min, mobile phase composition at 95% B was held constant. From 9–9.5 min, a linear gradient from 95% B to 5% B was applied, and from 9.5–16 min, the mobile phase flow rate was dropped to 0.2 mL/min and held at 5% B as wash steps were performed (see Section 4.3.1).

### 4.3. Development and Optimization

To determine the best chromatographic conditions for the 13 selected compounds, multiple combinations of trapping columns, analytical columns, and mobile phase solutions were evaluated using a mixed toxin standard with all 13 phycotoxins prepared in 100% methanol to each be a final concentration of 5 µg/L. Positive identifications of compounds were established through detection of the parent mass, confirmatory parent > daughter transitions, and reproducible retention times. Each peak was qualitatively evaluated for adherence to Gaussian and uniform peak shape. Quantitative evaluations were performed using signal intensities of peak height, and a color-coded table was produced providing information about these qualitative and quantitative observations.

To compare the effects of various column chemistries on analyte separation, various trapping columns and analytical columns were evaluated. Reverse-phase trapping columns (Oasis HLB Direct Connect HP Column, 2.1 × 30 mm, 20 µm, 80Å; XBridge BEH C18 Direct Connect HP Column, 2.1 × 30 mm, 10 µm, 130 Å; XBridge BEH C8 Direct Connect HP Column, 2.1 × 30 mm, 10 µm, 130 Å) and analytical columns (Acquity BEH C18, 2.1 × 50 mm, 1.7 µm, 130 Å; Acquity BEH C8, 2.1 × 50 mm, 1.7 µm, 130 Å) were purchased from Waters (Milford, MA, USA). VanGuard pre-columns matching the material of the analytical columns were also purchased from Waters.

In addition to traditional mobile phase solutions, UPLC-MS/MS with trap/ACD also utilizes loading and diluting phases. To determine the optimal conditions for loading, diluting, and mobile phases, multiple combinations of acidic, basic, and neutral solutions were evaluated for their relative effects on analyte separation, peak shape, and sensitivity [22]. Combinations (36) of columns, loading and diluting phases, and mobile phases, were tested. For these combinations, loading and diluting phases were prepared with 5% additive, and eluting phases were prepared with 0.5% additive.

Once the two best-performing methods were chosen from the 36 conditions, the amount of additive added to each solution was then varied to optimize conditions and create final methods. Signal intensities from injections of the same mixed-toxin standard were compared. Percent compositions for the loading phases were varied from 50 mM (0.2% *v*/*v*) to 1330 mM (5% *v*/*v*) formic acid for acidic, and from 6.7 mM (0.1% *v*/*v*) to 1300 mM (5% *v*/*v*) ammonium hydroxide for basic. For the mobile phases, acidic additive strengths ranged from 0.2% to 0.5% (*v*/*v*), while basic ranged from 0.1% to 0.5% (*v*/*v*). The minimum amount of additive tested for both basic and acidic loading and mobile phases was adopted from common literature values [19,31,33,38,39,40]. The addition of ammonium formate (2 mM) as a buffer to stabilize pH for the acidic solutions was also evaluated.

#### 4.3.1. Carryover Management

Due to the high-volume injections (50–1000 µL), carryover between samples was evaluated and various wash solutions were tested for their abilities to reduce carryover under the final methods. To evaluate for carryover, six 50-μL injections of a 100% methanol blank were run after one 50-μL injection of a 5 μg/L mixed-toxin standard prepared in 100% methanol. Peak areas from the blank run were compared to peak areas from the standard run. Carryover was calculated as *blank peak area/standard peak area* × *100*. Wash steps were prepared using mixtures of acetonitrile, methanol, isopropyl alcohol, acetone, and formic acid, and the timing and length of the wash steps was varied.

### 4.4. Method Performance Characteristics

The final methods were validated through a series of steps to confirm repeatability, establish method detection and quantification limits, determine the linearity range of standard curves, and confirm positive identification of each compound being evaluated. Parent > parent transitions were used during method performance characterization. Data generated using parent > parent transitions were later compared to parent > daughter transitions in SPATT extract (see Section 2.5).

#### 4.4.1. Repeatability

To evaluate repeatability, 7 consecutive injections of an 8 µg/L mixed-toxin standard in 100% methanol were run. Standard deviations were calculated using the peak areas for each compound. Percent relative standard deviations (%RSDs) for each compound were calculated as *100* × *standard deviation/average peak area.*

#### 4.4.2. Limits of Detection and Quantification

Limits of detection (LODs) were determined at two injection volumes, using two approaches: mathematically and by reporting the lowest concentration at which the signal to noise ratio (S/N) was greater than or equal to 3. To determine LODs mathematically, a mixed-toxin standard solution at a concentration 10× the estimated LOD for each compound was prepared in methanol. Repeated 50-μL injections (6) from a vial containing this standard were run, and the standard deviations of the peak areas for each compound were calculated. The LOD peak area for each compound was calculated as *3.14* × *standard deviation,* and the LOD concentration was calculated as *LOD peak area* × *sample concentration/average peak area.* Limits of quantification (LOQs) are reported as the lowest concentration at which S/N was greater than or equal to 10.

LODs were likewise determined for 1000 μL injections. A mixed-toxin standard solution was prepared in methanol at 10× the estimated LOD for a 1000 μL injection, calculated using the LOD data from the 50 μL injections. Repeated injections (6) were run, and LODs were calculated as above. A mixed-toxin standard solution at the estimated LOD for a 1000 μL injection was also prepared, and triplicate injections were run to confirm S/N ≥ 3.

#### 4.4.3. Linearity

Two standard curves, a low and a high concentration curve, were produced for each compound to assess linearity across multiple orders of magnitude. A series of 20 dilutions of mixed-toxin standards were prepared between 0.04 µg/L and 50 µg/L. In triplicate, 50 μL injections were run using the two methods finalized through method development, and two standard curves were produced to determine high-concentration and low-concentration ranges of linearity. To confirm linearity, squared correlation coefficient (R^2^) values must be >0.99.

### 4.5. SPATT Analysis

To determine whether the developed methods were acceptable to use as a screening tool for environmental samples, SPATT passive samplers (SPATTs) were deployed, extracted, and analyzed for the 13 phycotoxins included in this study using the two final methods. Twelve SPATTs were constructed using 3.15 g Diaion HP20 resin. In triplicate, SPATTs were deployed in three locations in the lower Chesapeake Bay region for 2 weeks in April 2018: a tidal river estuary (York River, salinity ca. 22), coastal bayside (Nassawadox, salinity ca. 18), and seaside Eastern Shore (Wachapreague, salinity ca. 32). Upon recoveries, SPATTs were frozen until extraction. A traditional SPATT extraction method was employed [23]; in brief, SPATTs were thawed, rinsed with ultrapure water, transferred and packed into empty glass solid phase extraction reservoirs, and extracted using 23 mL 100% methanol, (8:1 mL/g resin) at a flow rate no greater than 1 mL/min. Injections, 50 µL, of methanolic SPATT extracts were analyzed using the developed methods to quantify any endogenous phycotoxins. Results were normalized to ng toxin per gram SPATT resin per day.

A subsample of each extract was also analyzed after being spiked with a multi-toxin solution (final concentration of 5 µg/L per phycotoxin) to determine relative signal suppression or enhancement due to matrix effects. Results from the spiked SPATT extracts were compared to a 5 µg/L multi-toxin standard in 100% methanol. Possible signal enhancement or suppression, introduced by the sample matrices, was evaluated using the equation [41] *Matrix Effect* (%) *= 100* × *spiked extract peak area/spiked standard peak area*. For extracts in which endogenous compounds were already present before spiking, percent suppression or enhancement was calculated as *µg measured in spiked sample/(µg endogenous toxin + µg toxin added)×100*. KmTx 3 and GDA were not included in the spiking experiment due to the limited availability of purified material.

## Figures and Tables

**Table 1:** Summary [2,28,29] of 13 phycotoxins included in the development of a UPLC-MS/MS screening method

Phycotoxin Congener Abbreviation (Group)	Producer	Human Syndrome or Toxicity
GDA (goniodomins)	*Alexandrium monilatum*	Ichthyotoxic
OA; DTX1; DTX2 (okadaic acid and dinophysistoxins)	*Dinophysis* spp., *Prorocentrum lima*	Diarrhetic shellfish poisoning (DSP)
PTX2 (pectenotoxins *)	*Dinophysis* spp.	Acute toxicity in vertebrate model with i.p. injection
YTX (yessotoxin *)	*Protoceratium reticulatum*, *Gymnodinium catenatum*, *Pyrodinium bahamense*, *Gonyaulax* spp. *Lingulodinium polyedrum*	Acute toxicity in vertebrate model with i.p. injection
AZA-1; AZA-2 (azaspiracids)	*Amphidoma languida*, *Azadinium* spp.	Azaspiracid shellfish poisoning (AZP)
KmTx 3 (karlotoxins)	*Karlodinium* spp.	Ichthyotoxic
MC-LR; MC-YR; MC-RR (microcystins)	*Microcystis* spp., *Anabaena* spp., *Oscillatoria* spp. *Planktothrix* spp.	Hepatotoxic
PbTx-2 (brevetoxins)	*Karenia* spp.	Neurotoxic shellfish poisoning (NSP), ichthyotoxic

* Indicates a toxin group that is regulated in seafood products in the EU, but not in the US.

**Table 2:** Chromatographic conditions for the two final UPLC inlet methods: acidic and basic

Analyte	Trapping Column	Loading Conditions	Separation Column	Elution Conditions
MC-LR MC-YR MC-RR AZA1 AZA2 KmTx 3	XBridge BEH C18 130 Å 10 µm, 2.1 × 30 mm	H_2_O + 50 mM formic acid + 2 mM ammonium formate Isocratic for 3 min	Acquity BEH C18 130 Å 1.7 µm, 2.1 × 50 mm	**Acidic**** A: H_2_O + 50 mM formic acid + 2 mM ammonium formate B: ACN + 50 mM formic acid + 2 mM ammonium formate Linear gradient 5% to 95% B over 5 min Isocratic 95% B for 1 min
GDA PTX2PbTx-2YTXOA DTX1 DTX2	XBridge BEH C18 130 Å 10 µm, 2.1 × 30 mm	H_2_O + 6.7 mM NH_4_OH Isocratic for 3 min	Acquity BEH C18 130 Å 1.7 µm, 2.1 × 50 mm	**Basic**** A: H_2_O + 6.7 mM NH_4_OH B: ACN + 6.7 mM NH_4_OH Linear gradient 5% to 95% B over 5 min Isocratic 95% B for 1 min

**Table 3:** Results for the method performance characteristics of repeatability and limits of method detection and quantification using final two methods: acidic and basic. Limits of detection found in the literature from other studies using various LC-MS methods are presented for comparison.

	Literature		This Study							
Analyte	LOD LC-MS Methods (µg/L)	Reference	LOQ (S/N ≥ 10) 50 µL (µg/L)	LOD (S/N ≥ 3) 50 µL (µg/L)	%RSD 50 µL	LOD Calc. 50 µL (µg/L)	LOD Calc. 50 µL (pg on- column)	%RSD 1000 µL	LOD Calc. 1000 µL (µg/L)	LOD Calc. 1000 µL (pg on- column)
MC-RR	0.017 ^	[32]	0.31	0.13	3.81	0.07	3.5	3.66	0.007	7.5
MC-YR	0.043 ^	[32]	0.15	0.13	3.23	0.24	12	7.32	0.01	15
MC-LR	0.029 ^	[32]	0.15	0.13	3.10	0.25	13	5.80	0.01	12
AZA1	0.033	[33]	004	0.03	3.02	0.02	1.0	2.12	0.001	1.0
AZA2	0.070	[34]	0.04	0.03	2.56	0.01	0.5	3.11	0.001	1.5
GDA	2.34	[35]	1.98	0.60	2.43	0.39	19.5	2.45	0.019	19.2
KmTx3	4.0 *	[36]	1.39	0.97	5.64	0.64	32	5.64	0.05	54
OA	0.483	[33]	0.15	0.13	1.21	0.10	5.0	4.05	0.008	8.3
DTX1	0.030	[34]	0.15	0.13	2.30	0.11	5.5	5.87	0.01	12
DTX2	0.930	[20]	1.24	0.13	1.43	0.12	6.0	3.05	0.006	6.2
PTX2	0.048	[33]	0.04	0.03	1.89	0.04	2.0	3.91	0.004	3.7
YTX	0.336	[33]	1.24	0.50	1.19	0.14	7.0	4.27	0.03	34
PbTx-2	n.r.		0.15	0.13	7.18	0.16	8.0	7.39	0.03	15

^^^ Indicates when reported detection limit includes SPE concentration step, * indicates when reported on-column detection limit was converted to µg/L, S/N signal to noise ratio, %RSD percent relative standard deviation, LOQ limit of quantification, LOD limit of detection, calc. mathematically derived from six repeated injections, n.r. none reported.

**Table 4:** Concentrations of endogenous phycotoxins in Chesapeake Bay SPATT extracts determined using the parent > parent transition (P > P) and the parent > daughter transition (P > D) for each compound. The final optimized methods were utilized with a 50 µL injection volume.

	York		Nassawadox		Wachapreague	
	P > P (µg/L)	P > D (µg/L)	P > P (µg/L)	P > D (µg/L)	P > P (µg/L)	P > D (µg/L)
OA	34 ± 2.4	27 ± 2.3	21 ± 0.4	17 ± 0.3	13 ± 1.7	11 ± 1.2
PTX2	3.9 ± 0.7	3.5 ± 0.6	2.1 ± 0.04	1.9 ± 0.6	0.6 ± 0.1	0.6 ± 0.1
DTX1	6.1 ± 0.7	6.1 ± 0.7	2.8 ± 0.1	2.9 ± 0.2	1.0 ± 0.2	1.0 ± 0.2

**Table 5:** Signal enhancement (>100%) and signal suppression (<100%), ± standard deviation, observed in spiked extracts from triplicate SPATTs deployed in York River, Nassawadox, and Wachapreague analyzed using final two methods: acidic and basic. Values are reported using the equation: *Suppression or Enhancement* (%) *= 100* × *spiked extract peak area/spiked standard peak area.*

	Suppression (<100%) or Enhancement (>100%)		
	York (%)	Nassawadox (%)	Wachapreague (%)
MC-RR	26 ± 1	39 ± 3	38 ± 7
MC-LR	36 ± 0.4	50 ± 2	47 ± 4
MC-YR	32 ± 1	46 ± 2	44 ± 3
AZA1	33 ± 1	33 ± 2	43 ± 12
AZA2	49 ± 3	44 ± 3	52 ± 8
PbTx-2	146 ± 7	109 ± 13	123 ± 5
YTX	103 ± 2	132 ± 10	121 ± 18
DTX2	69 ± 0.8	81 ± 1	78 ± 3
OA *	101 ± 1	103 ± 0.7	101 ± 1
PTX2 *	57 ± 2	45 ± 0.8	36 ± 7
DTX1 *	98 ± 2	96 ± 3	91 ± 1

* calculated by difference due to endogenous phycotoxins present.

**Table 6:** Mass spectrometry parameters for 13 phycotoxins determined through direct infusion experiments

Analyte	Ionization Mode	Adduct	Molecular Ion *m/z*	Cone Voltage (V)	Dominant Transition Detected	Collision Energy (eV)
MC-RR	ESI+	2H+	520.0	30	520.0 > 135.1	30
MC-YR	ESI+	H+	1045.5	30	1045.5 > 135.1	85
MC-LR	ESI+	H+	995.5	30	995.5 > 135.1	85
AZA1	ESI+	H+	842.4	30	842.4 > 824.6	30
AZA2	ESI+	H+	856.4	30	856.4 > 838.6	30
KmTx 3	ESI+	Na+	1347.7	70	1347.7 > 937.7	80
GDA	ESI+	NH_4_+	786.5	30	786.5 > 139.0	40
OA	ESI−	−H	803.5	30	803.5 > 255.5	60
DTX1	ESI−	−H	817.5	30	817.5 > 113.0	70
DTX2	ESI−	−H	803.5	30	803.5 > 255.5	60
PTX2	ESI+	NH_4_+	876.6	30	876.6 > 841.5	30
YTX	ESI−	−2H	571.1	30	571.1 > 467.7	30
PbTx-2	ESI+	H+	895.4	40	895.4 > 877.3	20
